# Factors Associated With Sports Function and Psychological Readiness to Return to Sports at 12 Months After Anterior Cruciate Ligament Reconstruction: A Cross-sectional Study

**DOI:** 10.1177/03635465231192983

**Published:** 2023-09-08

**Authors:** Anna Cronström, Charlotte K. Häger, Kristian Thorborg, Eva Ageberg

**Affiliations:** † Department of Health Sciences, Lund University, Lund, Sweden; ‡ Department of Community Medicine and Rehabilitation, Umeå University, Umeå, Sweden; § Department of Orthopaedic Surgery, Copenhagen University Hospital, Copenhagen, Denmark; Investigation performed at Lund University, Lund, Sweden, and Umeå University, Umeå, Sweden

**Keywords:** anterior cruciate ligament, knee injury, physical function, psychology, patient-reported outcomes

## Abstract

**Background:**

Sports function and psychological readiness to return to sports (RTS) are important outcomes when evaluating rehabilitation after anterior cruciate ligament reconstruction (ACLR). It is, however, unclear which specific factors contribute most to these outcomes.

**Purpose:**

To determine associations between demographic characteristics, objective measurements of physical function, patient-reported outcome measure scores, sports-related function assessed with the Knee injury and Osteoarthritis Outcome Score (KOOS) Sport and Recreation subscale, and psychological readiness to RTS assessed with the Anterior Cruciate Ligament–Return to Sport after Injury (ACL-RSI) scale at 1 year after ACLR.

**Study Design:**

Cross-sectional study; Level of evidence, 3.

**Methods:**

At a mean of 12.5 ± 2.0 months after ACLR, 143 participants (50.3% female), with a mean age of 25.0 ± 5.7 years, were assessed for demographic characteristics, physical factors (hop performance, muscle strength, ankle and hip range of motion), and psychological factors (KOOS Pain and Symptoms subscales, Perceived Stress Scale, fear of reinjury) as well as the KOOS Sport and Recreation subscale and ACL-RSI scale. Backward linear regression models were used to evaluate factors associated with sports function and psychological readiness to RTS.

**Results:**

Lower isokinetic knee extension peak torque (limb symmetry index) (B = 18.38 [95% CI, 3.01-33.75]), lower preinjury activity level (B = 2.00 [95% CI, 0.87-3.14]), greater knee pain (B = 0.90 [95% CI, 0.70-1.10]), shorter time between injury and reconstruction (B = 0.16 [95% CI, 0.05-0.26]), and greater fear of reinjury (B = 0.11 [95% CI, 0.01-0.20]) were associated with a worse KOOS Sport and Recreation subscore (*R*^2^ = 0.683). A shorter hop distance (B = 0.15 [95% CI, 0.00-0.29]) was associated with a lower ACL-RSI score (*R*^2^ = 0.245).

**Conclusion:**

A combination of knee muscle strength, activity level, knee pain, timing of surgery, and fear of reinjury accounted for approximately 70% of the variation in sports function at 1 year after ACLR. In contrast, there was only 1 weak association between physical function and psychological readiness to RTS at this time point. Thus, factors associated with current sports function are much better known than features related to psychological readiness to RTS.

An anterior cruciate ligament (ACL) injury is common in sports and may lead to several physical and psychological consequences for the athlete, such as impaired muscle function,^1^ failure to return to sports (RTS),^6^ fear of movement and reinjury,^10,46^ and decreased quality of life.^20^ The rehabilitation period after ACL reconstruction (ACLR) is typically recommended to be 9 to 12 months,^58^ with adequate knee function and return to previous activity levels as the main goals.^37,44^ Yet, the risk of reinjury to either knee is quite high, with up to one-fourth of young athletes sustaining a secondary injury^65^ and the majority occurring within the first 2 years after RTS.^61^ To prevent secondary injuries and enhance safe RTS, athletes are advised to fulfill certain criteria, including both objective and patient-reported measures related to knee function, before returning to sports.^9^

The Knee injury and Osteoarthritis Outcome Score (KOOS), particularly its Sport and Recreation subscale, is an important measure of sports function when evaluating outcomes after ACLR with subsequent rehabilitation and RTS.^11,37^ Despite a plethora of knee injury management programs, sports function is still often reported to be impaired at 1 year after ACLR.^3,25^ In recent years, psychological factors such as a fear of reinjury and stress, and how these may influence movement patterns, have received increased attention as important aspects for reaching rehabilitation goals and making well-grounded RTS decisions.^7,38^

The Anterior Cruciate Ligament–Return to Sport after Injury (ACL-RSI) scale^63^ was developed to assess psychological effects on the RTS decision after ACLR. Lower psychological readiness to RTS has, indeed, been reported to be associated with both failure to RTS^5,62^ and a higher risk of a second ACL injury to either knee.^17^ Female patients seem to report both worse KOOS Sport and Recreation subscores and lower ACL-RSI scores than male patients at 12 months after ACLR.^2,64^ However, previous studies on contributing factors for sports function and psychological readiness to RTS include only a limited number of explanatory variables. Demographic, psychological, and/or functional factors that contribute to sports function and psychological readiness to RTS are, thus, not fully identified.

In cross-sectional studies, worse hop performance and worse movement quality have been demonstrated to be weakly to moderately related to lower scores on the KOOS Sport and Recreation subscale at 2 to 3 years after ACLR,^21,49^ whereas there were no associations between knee muscle strength at 3 years^21^ or sensory function at 5 months^15^ after ACLR and KOOS Sport and Recreation subscores. Furthermore, evidence for whether there is an association between isokinetic knee strength or hop performance and psychological readiness to RTS at 6 to 12 months after ACLR is inconsistent.^4,25,45,64^

To our knowledge, there are no studies on factors associated with sports function and/or psychological readiness to RTS that include a comprehensive set of relevant demographic characteristics, objective measurements of physical function, and subjective feelings of knee function around the time of expected RTS after ACLR. Several patient-reported outcome measures (PROMs), such as the KOOS^11^ and questionnaires on the fear of reinjury,^26^ as well as different measures of physical function are typically used for the evaluation of ACLR and rehabilitation outcomes.^58^ Hop performance and knee strength are important RTS criteria,^56^ and lower extremity and trunk strength as well as ankle and hip range of motion (ROM) are related to both altered movement patterns^14,36^ and the risk of lower extremity injuries.^42,59^ Identifying clinically relevant factors that are associated with sports function and psychological readiness may promote rehabilitation protocols that effectively target both physical and psychological aspects of sports function and RTS for these patients.

The aim of this exploratory cross-sectional analysis of data from an ongoing longitudinal project (clinical trial No. NCT04162613)^13^ was, thus, to determine possible associations between demographic characteristics, objective measurements of physical function, and PROM scores as well as sports function and psychological readiness to RTS assessed with the KOOS Sport and Recreation subscale and the ACL-RSI scale, respectively, at 1 year after ACLR.

## Methods

This study adheres to the Strengthening the Reporting of Observational Studies in Epidemiology guidelines (www.strobe-statement.org).

### Participants

**Figure 1.**
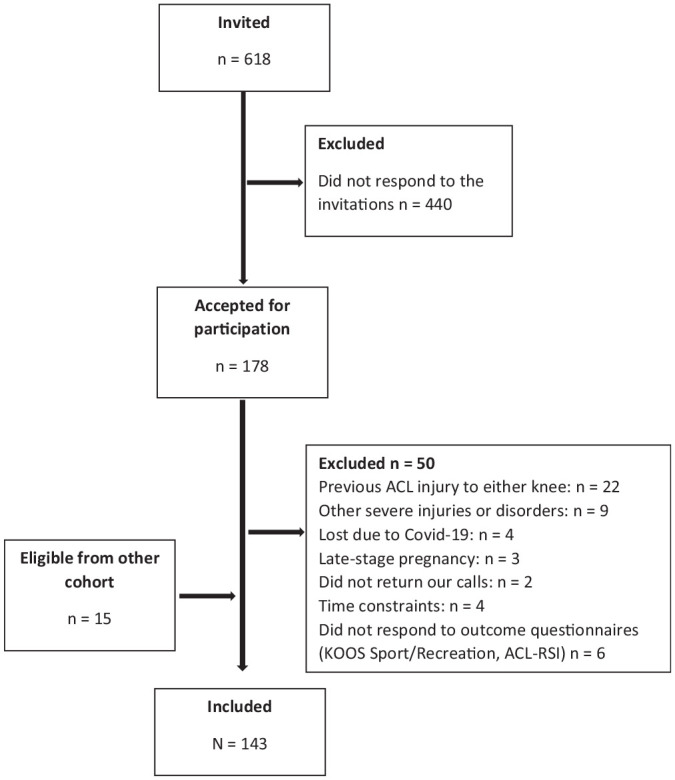
Flowchart of the inclusion process. Participants recruited between March 2018 and January 2022. ACL, anterior cruciate ligament; ACL-RSI, Anterior Cruciate Ligament–Return to Sport after Injury scale; KOOS, Knee injury and Osteoarthritis Outcome Score.

This study included 15 participants eligible from a previous cohort (data collected in 2016)^12^ and 128 participants recruited between 2018 and 2022, who were all part of the longitudinal project.^15^ Consequently, a total of 143 participants (n = 72 female) with a mean age of 25.0 ± 5.7 years were included (Table 1). Inclusion criteria were time of approximately 1 year after ACLR (range, 8-16 months), with or without associated injuries, and age of 15 to 35 years. Exclusion criteria were previous ACL injuries to the ipsilateral or contralateral knee, other diseases or disorders influencing functional performance (eg, hernia, neurological diseases), and not understanding the English or Scandinavian language (Figure 1). Participants with and without associated injuries, with different graft types, and within a range of 8 to 16 months after ACLR were included to reflect the clinical population, with these variables incorporated as possible explanatory factors for the outcomes in the current study. All participants provided written informed consent, and the study was approved by the Swedish Ethical Review Authority (Dnr 2016/319 and Dnr 2019-04037).

**Table 1:** Characteristics of Participants^*a*^

	Value
Age (N = 143), y	25.0 ± 5.7
Female sex (N = 143)	72 (50.3)
Body mass index (n = 133), kg/m^2^	24.3 ± 3.6
Family history of ACL injuries (n = 119)	24 (20.2)
Preinjury Tegner score (N = 143)	7 (5-9)
Current Tegner score (N = 143)	4 (2-7)
Return to same or higher activity level (n = 140)	49 (35.0)
Time between injury and reconstruction (n = 132), mo	9 (4-21)
Time since reconstruction (n = 108), mo	12.5 ± 2.0
Graft type (N = 143)	
Hamstring	113 (79.0)
Patella	17 (11.9)
Donor (graft type unknown)	1 (0.7)
NR	12 (8.4)
Associated injuries (n = 135)	105 (77.8)
Meniscus	91 (67.4)
Cartilage	28 (20.7)
Collateral ligament	37 (27.4)
Injury mechanism (N = 143)	
Contact	47 (32.9)
Noncontact	80 (55.9)
Other (trauma)	3 (2.1)
NR	13 (9.1)

a Data are presented as mean ± SD, n (%), or median (interquartile range). ACL, anterior cruciate ligament; NR, not reported.

### Data Collection

The participants were assessed with PROMs and a comprehensive test battery for physical function including ankle and hip ROM, isokinetic and isometric muscle strength, and hop performance at 1 year after ACLR. A warm-up of 5 minutes of stationary cycling preceded physical testing. Data for all questionnaires were collected via an electronic data capture tool (REDCap; hosted by Lund University).^24^ A full description of all included measures has been previously published,^12,13^ and a brief description is provided in this article.

### Explanatory Variables

#### Patient Characteristics

The following patient factors were collected: age (derived from the Swedish personal identity number), date of surgery (derived from surgical records), body weight and height (assessed with anthropometric measures), sex, time between injury and surgery, family history of ACL injuries, concomitant injuries, graft type, and injury mechanism (all self-reported; no cross-checking was performed in the patients’ medical records). The Tegner activity scale,^55^ with scores from 1 to 10 (1 = walking on flat surface and 10 = elite-level participation), was used to assess preinjury and current activity levels.

#### Physical Function

##### Ankle and Hip ROM

Active ankle dorsiflexion (in degrees) was assessed using a plastic goniometer, as previously described,^47^ with the participant in a standing position with the knee against the wall. The participant was asked to slide the foot backward as far as possible with the knee maintaining contact with the wall and the heel maintaining contact with the floor. The mean of 3 measurements was used in statistical analysis. Passive hip external rotation and internal rotation were assessed using a digital inclinometer (Commander Echo; JTECH Medical), with the participant in a seated position with the hip and knee joints in 90° of flexion. Maximum passive external rotation and internal rotation were recorded when the assessor visually observed a lateral pelvic tilt. Total ROM (external rotation + internal rotation) was then calculated. The mean total ROM of 2 trials was used in analysis.^48^ High intraobserver and interobserver reliability have previously been reported for these measures (intraclass correlation coefficient [ICC] = 0.65-0.99).^47,48^

##### Isometric Muscle Strength

A handheld dynamometer (Commander PowerTrack II; JTECH Medical) was used to assess isometric peak force (in newtons) of the knee (flexion, extension), hip (extension, abduction, external rotation), and trunk (side bridge), as previously described.^12^ Before strength testing, the lever arm (in meters) was measured from the joint axis of rotation to the point of application of the force transducer for each test. For the side bridge, the distance between the acromion and the lateral malleolus was used for normalization. A belt was used to fixate the dynamometer, and the participants were asked to push with maximum effort against the dynamometer for 5 seconds during all tests. The peak value of 3 trials (in newton meters) was calculated by dividing the peak force value with the corresponding lever arm and then normalizing to body weight (in newton meters per kilogram) for analysis. We have previously reported good interobserver and intraobserver reliability for these measurements (ICC = 0.62-0.95).^12^

##### Isokinetic Muscle Strength

Isokinetic concentric peak torque during knee extension and flexion was measured at 60 deg/s with a dynamometer (Biodex Medical Systems). The maximum of 5 trials was recorded. The limb symmetry index (LSI; [injured leg/noninjured leg] × 100) for normalized peak torque (in newton meters per kilogram) for knee extension and flexion was used in analysis. High interobserver reliability has previously been reported for the Biodex device (ICC = 0.88-0.92).^18^

##### Hop Tests

Overall, 2 well-established and reliable (ICC = 0.93-0.95) hop tests were administered: the side hop and the single-leg hop for distance (SLHD).^23^ For the side hop, the participants jumped laterally/medially over 2 strips of tape, 40 cm apart. The test was performed once on each leg, and the maximum number of jumps in 30 seconds was recorded. For the SLHD, the participant was asked to jump as far as possible, taking off and landing on the same leg. The distance (in centimeters) from the toe at push-off to the heel in the landing position was assessed, and the longest hop distance obtained from 3 trials was used for analysis.

#### Patient-Reported Measures

Data from the following validated questionnaires were obtained: the KOOS Pain and Symptoms subscales.^11^ The KOOS includes questions that are assessed on a 0-to-100 scale in which 0 indicates poor knee-related function and 100 indicates excellent knee-related function. The fear of reinjury was evaluated by question 7 on the ACL-RSI scale (0 = worst to 100 = best): “Are you fearful of reinjuring your knee by playing your sport?” The Perceived Stress Scale^54^ was also used to evaluate the participants’ self-perceived stress.

### Outcomes

The validated and reliable (ICC = 0.85) KOOS Sport and Recreation subscale^11^ was used as a measure of sports function. The ACL-RSI scale,^32^ with scores from 0 to 100 (0 indicates low readiness, and 100 indicates high readiness), was used to assess psychological readiness to RTS. High test-retest reliability has been reported for this measurement (ICC = 0.89).^32^

### Statistical Analysis

SPSS (Version 28; IBM) was used for all statistical calculations. The normal distribution of original data and residuals was investigated by a visual inspection of histograms and Q-Q plots and the interpretation of skewness and kurtosis. All data were normally distributed (ie, data were symmetrical around the mean), except for time between injury and reconstruction. The independent *t* test (continuous data), Spearman rank correlation coefficient (ordinal, nonnormally distributed data), and Pearson correlation coefficient (continuous data) were used, as appropriate, to investigate any associations between participants’ characteristics, physical function variables, and PROM scores as well as ACL-RSI and KOOS Sport and Recreation scores. As it was part of the ACL-RSI scale and, thus, highly correlated with the ACL-RSI score, the fear of reinjury was only included in analysis for the KOOS Sport and Recreation subscale. Given that most participants had undergone ACLR with a hamstring tendon graft, graft type was excluded from further analysis. All variables that had a statistically significant association with ACL-RSI and/or KOOS Sport and Recreation scores were then incorporated in separate backward linear regression models, with demographic, physical function, and/or PROM factors as independent variables and ACL-RSI and KOOS Sport and Recreation scores as dependent variables (1 model for each outcome). A variance inflation factor <4 was used to assess potential collinearity between the included independent factors. Values from the participant's injured leg were used for all physical function variables, with the exception of isokinetic knee strength, in which the LSI was used. A *P* value ≤.05 was considered statistically significant. Because this was an explorative study, no adjustments for multiple comparisons were executed.^8^

## Results

The 15 participants included from the previous cohort from 2016^12^ did not undergo isokinetic muscle strength or hip ROM testing. Family history and perceived stress were also not assessed, consequently reducing the sample size for these specific variables. Because of the COVID-19 pandemic, 7 participants were not able to undergo the tests of physical function and were, thus, only included in the analysis for PROMs. In addition, 7 participants declined to perform the side hop, and 1 participant declined to perform the SLHD. Descriptive data for all variables of objective function and PROMs are presented in Appendix Table A1 (available in the online version of this article).

### Univariable Analysis

The following variables were significantly associated with the KOOS Sport and Recreation subscore and were included in the regression model: age; preinjury Tegner score; time between injury and surgery; hip ROM; isometric hip external rotation, hip extension, hip abduction knee flexion, knee extension, and side bridge peak torque; isokinetic knee extension and knee flexion peak torque (LSI); SLHD; side hop; KOOS Pain and Symptoms subscores; and fear of reinjury. The corresponding variables for the ACL-RSI score were as follows: time since reconstruction, hip ROM, isokinetic knee extension peak torque (LSI), SLHD, side hop, KOOS Pain subscore, and perceived stress (Table 2).

**Table 2:** Univariable Associations Between Factors and ACL-RSI and KOOS Sport and Recreation Scores^*a*^

	KOOS Sport and Recreation		ACL-RSI	
	Mean ± SD or Correlation Coefficient	*P* Value	Mean ± SD or Correlation Coefficient	*P* Value
Sex (n = 143)		.076		.462
Female	58.5 ± 24.3		44.2 ± 24.9	
Male	66.3 ± 27.2		47.3 ± 24.5	
Age (N = 143)	***r* = −0.200**	**.017**	*r* = −0.146	.083
Body mass index (n = 133)	*r* = −0.080	.360	*r* = 0.123	.161
Family history of ACL injuries (n = 119)		.530		.469
Yes	66.9 ± 22.3		50.1 ± 28.0	
No	63.3 ± 25.7		45.9 ± 24.9	
Preinjury Tegner score (N = 143)	***r*_s_ = 0.218**	**.009**	*r*_s_ = 0.073	.341
Time between injury and reconstruction (n = 132)	***r*_s_ = 0.182**	**.036**	*r*_s_ = −0.054	.543
Time since reconstruction (N = 108)	*r* = 0.129	.126	***r* = 0.196**	**.019**
Associated injuries (n = 135)		.165		.058
Yes	61.7 ± 25.0		43.6 ± 24.1	
No	69.0 ± 24.8		53.4 ± 26.3	
Injury mechanism (n = 143)		.195		.467
Contact	66.4 ± 25.6		48.1 ± 24.2	
Noncontact	61.4 ± 24.7		44.7 ± 25.9	
Range of motion				
Ankle (n = 134)	*r* = 0.100	.250	*r* = −0.450	.610
Hip (n = 118)	***r* = −0.217**	**.018**	***r* = −0.282**	**.002**
Isometric peak torque (n = 134)				
Hip external rotation	***r* = 0.306**	**<.001**	*r* = 0.132	.130
Hip extension	***r* = 0.240**	**.005**	*r* = 0.104	.233
Hip abduction	***r* = 0.172**	**.042**	*r* = 0.019	.824
Knee extension	***r* = 0.320**	**<.001**	*r* = 0.137	.116
Knee flexion	***r* = 0.285**	**<.001**	*r* = 0.186	.062
Side bridge	***r* = 0.194**	**.025**	*r* = 0.034	.697
Isokinetic peak torque (LSI) (n = 117)				
Knee extension	***r* = 0.335**	**<.001**	***r* = 0.332**	**<.001**
Knee flexion	***r* = 0.194**	**.036**	*r* = 0.158	.148
Hop performance				
SLHD (n = 135)	***r* = 0.492**	**<.001**	***r* = 0.279**	**.001**
Side hop (n = 129)	***r* = 0.463**	**<.001**	***r* = 0.291**	**<.001**
KOOS (N = 143)				
Pain	***r* = 0.677**	**<.001**	***r* = 0.330**	**<.001**
Symptoms	***r* = 0.221**	**.008**	*r* = 0.124	.141
Perceived Stress Scale (n = 128)	*r* = −0.120	.177	***r* = −0.176**	**.047**
Fear of reinjury (N = 143)	***r* = 0.291**	**<.001**	NA	NA

a Bold values indicate statistically significant associations. *r* = Pearson correlation coefficient; *r*_s_ = Spearman rank correlation coefficient. ACL, anterior cruciate ligament; ACL-RSI, Anterior Cruciate Ligament–Return to Sport after Injury; KOOS, Knee injury and Osteoarthritis Outcome Score; LSI, limb symmetry index; NA, not applicable; SLHD, single-leg hop for distance.

### Multivariable Analysis

The regression model revealed that lower isokinetic knee extension peak torque (LSI) (B = 18.38; *P* = .020), lower preinjury Tegner score (B = 2.00; *P* < .001), greater knee pain (B = 0.90; *P* < .001), shorter time between injury and reconstruction (B = 0.16; *P* = .005), and greater fear of reinjury (B = 0.11; *P* = .025) were associated with worse KOOS Sport and Recreation subscores (*R*^2^ = 0.683). A shorter SLHD (B = 0.15; *P* = .049) was the only variable with a statistically significant association with lower ACL-RSI scores (*R*^2^ = 0.245) (Table 3). No collinearity between any of the independent variables was observed (variance inflation factor ≤1.52).

**Table 3:** Variables Associated With KOOS Sport and Recreation and ACL-RSI Scores^*a*^

		B	SE	95% CI	β	*P* Value	*R* ^2^ (Adjusted *R*^2^)
KOOS Sport and Recreation							0.683 (0.664)
Preinjury Tegner score		2.00	0.57	0.87 to 3.14	0.21	<.001	
Time between injury and reconstruction		0.16	0.06	0.05 to 0.26	0.16	.005	
Isokinetic knee extension peak torque (LSI)		18.38	7.75	3.01 to 33.75	0.15	.020	
SLHD		0.15	0.09	−0.03 to 0.33	0.12	.090	
KOOS Pain		0.90	0.10	0.70 to 1.10	0.58	<.001	
Fear of reinjury		0.11	0.05	0.01 to 0.20	0.14	.025	
ACL-RSI							0.245 (0.213)
Time since reconstruction		2.56	1.34	−0.11 to 5.22	0.17	.060	
Isokinetic knee extension peak torque (LSI)		22.77	12.42	−1.86 to 47.40	0.18	.070	
SLHD		0.15	0.07	0.00 to 0.29	0.21	.049	
KOOS Pain		0.31	0.17	−0.03 to 0.64	0.19	.072	

a ACL-RSI, Anterior Cruciate Ligament–Return to Sport after Injury; KOOS, Knee injury and Osteoarthritis Outcome Score; LSI, limb symmetry index; SLHD, single-leg hop for distance.

## Discussion

In this explorative study, we found that lower knee extension strength, lower preinjury activity levels, greater knee pain, shorter time between injury and ACLR, and greater fear of reinjury all contributed, to a high extent, to worse sports function at 1 year after ACLR. A shorter SLHD was the only variable associated with lower psychological readiness to RTS at this time point, indicating that the variation in ACL-RSI scores may be explained by factors other than those included in this study.

Identifying factors that may improve self-reported sports function and psychological readiness to RTS is crucial because these are important outcomes to evaluate the rehabilitation process and may aid in decision-making when an athlete is ready to RTS.^37^ We investigated the associations between several seemingly important factors through a comprehensive test battery of physical function, the collection of patient characteristics, and PROMs, including pain and psychological aspects, in relation to sports function around the time of RTS. Interestingly, reduced isokinetic knee extension strength compared with the noninjured leg was the only measurement of physical function that was associated with worse sports function in the multivariable model. The result is in line with a study by Zwolski et al,^66^ reporting those with lower isokinetic knee extension peak torque (LSI) to have worse self-reported knee function, evaluated with the International Knee Documentation Committee score, at the time of RTS in patients after ACLR. Quadriceps strength is a significant contributor to knee joint stability,^22,29^ and greater quadriceps strength may also be related to better movement quality^34,52^ and better functional performance,^27^ factors that may be particularly important for self-reported sports function in the cohort of patients included in this study.

Other factors associated with sports function in the current study were demographic characteristics (preinjury activity level and timing of ACLR), knee pain, and psychological aspects. Those with a lower preinjury activity level may RTS at a lower level than those active at a higher level,^31^ which may be a reason for the association between lower preinjury activity levels and worse sports function. Less time between injury and surgery was associated with worse sports function. Although early reconstruction may increase the risk of subsequent ACL injuries to the contralateral knee,^16^ early reconstruction has previously been linked to a higher activity level after ACLR^19^ and better postoperative outcomes compared with delayed surgery.^28,30^ Early/acute reconstruction (within 1 week) has been reported to be related to an increased risk of arthrofibrosis,^51^ which potentially could affect outcomes. None of the participants in the current study, however, underwent acute ACLR. The reasons for the result in the present study are, thus, not clear and need further research.

Psychological factors are reported to be important for successful rehabilitation and RTS.^7^ The fear of reinjury specifically may decrease patients’ adherence to rehabilitation protocols,^60^ alter muscle activation patterns during activity,^38^ and be an important reason for an athlete's not returning to previous physical activity.^50^ In this study, we found that the fear of reinjury was associated with worse self-reported sports function, further highlighting psychological factors as crucial to consider in the rehabilitation process.

A shorter hop distance and lower LSI during the SLHD have previously been linked to lower psychological readiness to RTS at 1 year after ACLR.^25,64^ Although we included several different measures of physical function in addition to hop performance, such as muscle strength and ROM, along with demographic characteristics and PROMs, a shorter SLHD was the only variable that was associated with a lower ACL-RSI score in the multivariate model. Thus, this result confirms that the SLHD may be a relevant measure when evaluating psychological readiness to RTS. However, given the cross-sectional design of the current study, we do not know if jump ability affects patients’ psychological readiness to RTS or if they, in fact, jump a shorter distance because of psychological aspects, such as, for example, the fear of reinjury. Moreover, hop distance only contributed to 24% of the variation in the ACL-RSI score, indicating that there are several factors not captured by the variables included in this study that may be important for psychological readiness after ACLR. Nagelli et al^41^ reported reduced frontal-plane knee motion and increased frontal-plane hip motion during a single-leg drop landing task to be associated with lower scores on the ACL-RSI scale. Common impairments to the neurological system after an ACL injury, such as muscle inhibition, altered muscle activation patterns, decreased knee extension, and joint instability,^33^ were also not investigated in the current study. Movement quality/strategies, muscle activation amplitude, and joint stability during dynamic tasks as well as knee extension deficits may, thus, be other factors that could be explored further as contributors to psychological readiness.^41^

Another reason for the low association between the factors included in the current study may be related to the fear of reinjury. Because the fear of reinjury is included as a specific question on the ACL-RSI scale and, as such, is highly correlated with the total score, we did not include this variable in the analysis of contributing factors for psychological readiness to RTS. However, a recent network analysis of the psychological contributors to the ACL-RSI score concluded that the fear of reinjury and being relaxed about playing sports explained most of the variation in psychological readiness to RTS at 1 year after ACLR.^35^ The fear of reinjury, potentially along with other psychological factors not explored in the current study, such as motivation and knee confidence,^43,53,57^ may, therefore, be the primary drivers of psychological readiness to RTS, whereas physical function may contribute to a lesser extent.

This study has some limitations. Approximately 75% of eligible patients did not respond to the invitation to participate in the study (see Figure 1), which may have introduced selection bias. Although the KOOS Sport and Recreation subscores reported at 1 year in the current study correspond to those in the Swedish knee ligament registry,^3^ the reported ACL-RSI scores were considerably lower than those reported in other studies.^39,64^ The reason for the low psychological readiness to RTS in the current cohort is not clear, and we cannot rule out that this may have influenced the results. Furthermore, this was an explorative study including data from an ongoing longitudinal project. There was, thus, no a priori sample size calculation performed, and the study was powered for the original research purpose. In addition, given the exploratory design, no corrections for multiple comparisons were made.^8^ In regression analyses, it is typically recommended to include ≥10 participants for each independent variable in the model. Although this assumption was reached for the ACL-RSI scale (7 variables and 143 participants), we included 17 variables in the regression model for the KOOS Sport and Recreation subscale. Thus, we cannot exclude that some of the findings, especially in relation to sports function, may be caused by chance, and further studies are, therefore, needed to confirm our results. Injury-related data such as graft type and associated injuries were self-reported and may be affected by recall bias. A vast majority of the ACLR procedures in the current study were performed using a hamstring tendon graft. A recent network meta-analysis reported differences in joint stability, PROM scores, and RTS rates between different graft types (hamstring and patella),^40^ and, thus, we cannot generalize the results to those who have undergone reconstruction using a different graft. Along the same lines, we only included patients who had undergone ACLR with a time of 1 year after surgery, and we cannot generalize our findings to those with an ACL-deficient knee or other time interval since ACLR. It may also be argued that patients with and without associated injuries and a different duration between injury and ACLR (range, 8-16 months) may constitute different cohorts with different functional outcomes. We did include associated injuries and time since reconstruction as explanatory variables in our analysis, and neither variable was associated with sports function or psychological readiness to RTS. Furthermore, regression analysis only indicates whether a greater or smaller value of the explanatory variable is associated with outcomes, not if a specific cutoff value is associated with the outcome. Finally, this study had a cross-sectional design, and we cannot draw any conclusions on causal relationships.

## Conclusion

A combination of knee muscle strength, activity level, knee pain, timing of surgery, and fear of reinjury accounted for approximately 70% of the variation in sports function at 1 year after ACLR. In contrast, there was only 1 weak association between physical function and psychological readiness to RTS at this time point. Thus, factors associated with current sports function are much better known than features related to psychological readiness to RTS, at least other than those assessed in the present study.

## Supplemental Material

sj-pdf-1-ajs-10.1177_03635465231192983 – Supplemental material for Factors Associated With Sports Function and Psychological Readiness to Return to Sports at 12 Months After Anterior Cruciate Ligament Reconstruction Click here for additional data file. Supplemental material, sj-pdf-1-ajs-10.1177_03635465231192983 for Factors Associated With Sports Function and Psychological Readiness to Return to Sports at 12 Months After Anterior Cruciate Ligament Reconstruction by Anna Cronström, Charlotte K. Häger, Kristian Thorborg and Eva Ageberg in The American Journal of Sports Medicine
