# Supplementary material for: Factors Associated With Sports Function and Psychological Readiness to Return to Sports at 12 Months After Anterior Cruciate Ligament Reconstruction: A Cross-sectional Study
Source: Am J Sports Med. 2023 Sep 8;51(12):3112–20. doi: 10.1177/03635465231192983 (PMC10543957; doi:10.1177/03635465231192983)
Supplement: sj-pdf-1-ajs-10.1177_03635465231192983 – Supplemental material for Factors Associated With Sports Function and Psychological Readiness to Return to Sports at 12 Months After Anterior Cruciate Ligament Reconstruction [file sj-pdf-1-ajs-10.1177_03635465231192983.pdf]

Factors associated with sports function and psychological readiness to return to sport 12 months after ACL reconstruction: A cross-sectional study

Appendix

Table A1. Descriptive values for the predictors (physical function, PROMs) and outcomes

**Predictor**	**Mean (sd)**
Range of Motion (ROM)	
Ankle ROM (degrees) (n=134)	39.4 (5.1)
Hip ROM (degrees) (n=118)	70.3 (12.1)
Isometric peak torque (N·m/kg) (n=134)	
Hip external rotation	0.61 (0.18)
Hip extension	1.24 (0.25)
Hip abduction	1.67 (0.43)
Knee extension	1.82 (0.56)
Knee flexion	0.61 (0.21)
Side-bridge	4.99 (1.97)
Isokinetic peak torque (LSI) (n=117)	
Knee extension	86.7 (20.1)
LSI ≥90 n (%)	50 (43)
Knee flexion	90.2 (13.7)
LSI ≥90 n (%)	56 (48)
Hop performance	
SLHD (cm) (n=133)	114.5 (37.1)
Side-hop (n) (n=127)	35 (17.7)
KOOS (n=143)	
Pain	80.0 (16.8)
Symptom	61.4 (13.2)
Perceived stress scale (n=128)	19.3 (4.2)
Fear of re-injury (n=143)	39.1 (31.1)
**Outcomes** (n = 143)	
KOOS sport/recreation	62.4 (26.0)
ACL-RSI	45.7 (24.7)

PROMs = Patient Reported Outcome Measures, ACL-RSI = Anterior Cruciate Ligament Return to Sport after Injury scale, KOOS = Knee injury and Osteoarthritis Outcome Score, BMI = Body Mass Index, Kg = Kilogram, LSI = Limb Symmetry Index, SLHD = Single Leg Hop for Distance, Cm = centimeter
